# HO‐1 regulates the function of Treg: Association with the immune intolerance in vitiligo

**DOI:** 10.1111/jcmm.13723

**Published:** 2018-07-05

**Authors:** Qian Zhang, Tingting Cui, Yuqian Chang, Weigang Zhang, Shuli Li, Yuanmin He, Bing Li, Ling Liu, Gang Wang, Tianwen Gao, Chunying Li, Zhe Jian

**Affiliations:** ^1^ Department of Dermatology Xijing Hospital Fourth Military Medical University Xi'an China; ^2^ Department of Dermatology The Affiliated Hospital of Southwest Medical University Luzhou China

**Keywords:** Hemin, HO‐1, IL‐10, Treg, vitiligo

## Abstract

In vitiligo, cutaneous depigmentation is accompanied by increased T cell cytolytic activity targeting melanocytes, indicating that autoimmune tolerance is disrupted. The inhibited amount and function of Tregs have been indicated to be involved in the autoimmune intolerance in vitiligo, however, with the conclusion still controversial and the involved mechanism unknown. In this study, we explored the molecular and cellular alterations accounting for the impaired Treg response in vitiligo. Our results showed that the amount of Tregs was drastically reduced in peripheral blood of active vitiligo patients. Furthermore, the immunoregulatory function of Tregs was attenuated, with lower expression of CTLA4, IL‐10 and TGF‐β. Moreover, the expression of HO‐1, a functional modulator of Tregs, was decreased in vitiligo Tregs, and the concentrations of HO‐1 metabolites, including bilirubin, CoHb and iron, were correspondingly decreased in serum of vitiligo patients. In addition, we treated the Tregs from vitiligo patients with Hemin, an agonist of HO‐1, and found that enhanced HO‐1 expression restored the function of Tregs by up‐regulating IL‐10 expression. Our study demonstrates the essential role of HO‐1 in the impaired Treg response in vitiligo and indicates the potential of HO‐1 as a therapeutic target in vitiligo management.

## 1 INTRODUCTION

Vitiligo is a chronic skin disease with a worldwide incidence rate of approximately 0.5%‐1.0%.^1^ The loss of melanocytes in the epidermis is the key process in the development of vitiligo, which is the direct reason for the formation of patchy depigmentation on the skin of vitiligo patients. Recent studies have demonstrated that overactive T‐lymphocytes mediate the destruction of melanocytes in the pathogenesis of vitiligo,^2^, ^3^ indicating that autoimmune tolerance is disrupted in the disease.

Regulatory T cells (Tregs) contribute to immune homoeostasis by enforcing negative regulation on other immune cells, such as suppressing the activation and expansion of autoreactive CD4^+^ and CD8^+^ T cells. In 2010, Klarquist J et al^4^ first reported that the percentage of Tregs among skin infiltrating T cells drastically reduces in non‐lesional, perilesional and lesional skin from vitiligo patients, which was further supported by many other subsequent researches.^5^, ^6^ Moreover, increasing the abundance of Tregs by CCL22 overexpression can reduce depigmentation in two mouse models of vitiligo,^7^ indicating that replenishing Tregs can repair the disrupted autoimmune tolerance and is a promising treatment for vitiligo.

Recently, it is reported that Tregs are suppressed in vitiligo not only on their amounts but also on their function. The polymorphisms of several immunosuppressive genes expressed in Tregs including transcription factor Forkhead box P3 (Foxp3),^8^ interleukin‐10 (IL‐10)^9^ and transforming growth factor β (TGF‐β)^10^ are associated with susceptibility to vitiligo. Moreover, the function of Tregs has been shown impaired in vitiligo patients, with weaker suppressive effect of Tregs on autologous CD8^+^T cells^4^ and decreased expression level of Foxp3 and cytotoxic T lymphocyte antigen‐4 (CTLA‐4) that induces the anergy of effector T cells.^6^ Therefore, amending the impaired function of Tregs is another way to reconstruct the autoimmune tolerance and may be applied to the treatment for vitiligo.

Heme oxygenases‐1 (HO‐1) is a rate‐limiting enzyme that degrades heme into biliverdin, yielding carbon monoxide (CO) and free iron.^11^ HO‐1 exerts antioxidant, anti‐apoptotic and immune‐modulating functions, leading to overall cytoprotective effect on mammalian cells. HO‐1 has been proved to modulate the function of Tregs.^12^ For instance, enhanced HO‐1 is necessary for the regulation of Tregs on the balance of Th1/Th2 response in Spesis,^13^ while the blockage of HO‐1 abrogates the protective effect of Treg on immune homoeostasis during murine pregnancy.^14^ Given that the level of HO‐1 is significantly decreased in vitiligo,^15^ we hypothesized the deficiency of HO‐1 may be a key reason for the impaired function of Tregs in vitiligo.

To test this hypothesis, we recruited 51 vitiligo patients and 51 age‐ and sex‐matched healthy controls. We compared their Tregs to CD4^+^ T cells ratio in peripheral blood, and evaluated the association between the Tregs to CD4^+^ T cells ratio and disease activity or lesional areas. Besides, we analysed the immunoregulatory ability of Tregs towards effector T cells and the proliferative ability of Tregs. Furthermore, the key immunosuppressive molecules involved in the function of Tregs were also tested, and at last we attempted to restore the Treg function through regulating HO‐1 expression.

## 2 MATERIALS AND METHODS

### 2.1 Patients

All patients and donors were recruited from Xijing Hospital, Fourth Military Medical University. All vitiligo patients did not receive any systemic treatment including immunosuppressive agents and phototherapy for at least 3 months prior to the procedures. Patients with other autoimmune diseases were excluded from this study. Information on demographics, and other characteristics were obtained by questionnaires. Patients whose lesional skin area progressed in the past 1 month were defined as active patients, and others were defined as stable patients. All the subjects voluntarily agreed to participate in this study and signed informed consent forms. Each of them donated 20 mL of blood, which was collected in heparinized tubes for further separation of PBMCs. This study was approved by the ethics review board of Fourth Military Medical University and was conducted according to the principles of Helsinki Declaration. The characteristics of the participants were summarized in Table 1.

**Table 1:** Demographic and clinical characteristics of study participants

	Vitiligo patients	Healthy controls
Range of age in years, mean (SD)	13‐66, 36.35 (14.35)	15‐67, 35.55 (14.28)
Gender, n (%)		
Male	34 (66.67)	34 (66.67)
Female	17 (33.33)	17 (33.33)
Range of disease duration in months, mean (SD)	1‐600, 129.29 (130.76)	NA
Disease activity of vitiligo, n (%)		NA
Stable (No progress in past 1 mo)	20 (39.22)	NA
Active (rapidly progress within 1 mo)	31 (60.78)	NA
Lesional skin (n% of BSA)		
<1%	4 (7.84)	NA
1%‐5%	21 (41.18)	NA
5%‐50%	21 (41.18)	NA
>50	5 (9.80)	NA

NA, not applicable; SD, standard deviation; BSA, body surface area.

### 2.2 Cell isolation of PBMCs, Tregs and CD8^+^ T cells

PBMCs were isolated from peripheral blood by Ficoll gradient centrifugation (DAKEWE, Shenzhen), CD4^+^CD25^high^ Treg cells and CD8^+^ T cells were sorted from PBMCs by flow cytometry through signalling of appropriate antibodies: anti‐CD4‐FICT (Biolegend), anti‐CD25‐APC (Biolegend) and anti‐CD8‐Percp‐cy5.5 (Biolegend), respectively. The antibodies were incubated at 4°C for 30 minutes in the dark. Flow cytometric analyses were performed using Fluorescence Activated Cell Sorter (FACS) Canto II (BD Biosciences, Heidelberg, Germany), and the results were analysed with BD FACSDiva Software version 6.1.2. For detection of surface molecules of CD4^+^CD25^high^ regulatory cells, PBMCs were incubated with the appropriate mAb: anti‐CD4‐FITC (eBioscience) and anti‐CD25‐PE (eBioscience). For intracellular staining including the staining of Foxp3, HO‐1, IL‐10, LAP and CTLA‐4, cells were fixed, permeabilized and stained according to the manufacturer's instruction. The following mAb were used: anti‐Foxp3‐APC (eBioscience), anti‐HO‐1‐PE (Abcam), anti‐IL‐10‐PE (eBioscience), anti‐LAP‐PerCP (eBioscience) and anti‐CTLA‐4‐PE (Biolegend).

### 2.3 Regulatory activity assay

To test the regulatory activity of Tregs, 3.5 × 10^4^ CD8^+^ T cells isolated from healthy subjects were firstly labelled with carboxyfluorescein diacetate, succinimidyl ester (CFSE 2 μmol/L, Invitrogen, Life Technologies) and the labelled CD8^+^T were seeded in 96‐well plates in 200 μL RPMI medium containing rhIL‐2 (50 ng/mL, Peprotech, Rocky Hill, NJ); then, the Treg cells isolated from patients or another healthy controls were added into each well at a mixing ratio of 1:1. After co‐culturing for 7 days, the proliferation rate of CD8^+^ T cells was analysed by FACS through signalling of CFSE. The results were calculated by FlowJo software version 7.6.1 (Tree Star, Ashland, OR). The reduction in the proliferation rate of CD8^+^ T cell was used to measure the inhibitory capacity of Tregs. The calculation method is as follows: the proliferation ratio of CD8^+^T cell minus the proliferation ratio of CD8^+^T cell with VP‐Treg or HC‐Treg under the same conditions (with or without Hemin).

### 2.4 Proliferation assays in vitro

To test the proliferating ability of Tregs, Tregs were isolated from the PBMCs by flow cytometry and then labelled with CFSE at room temperature for 1 hour. The anti‐CD3/28 antibodies were pre‐coated in the U‐bottom plate, followed by addition of rhIL‐2 (50 ng/mL, Peprotech, Rocky Hill, NJ). After incubation for 7 days, the cells were harvested and the proliferation rates of Tregs were analysed by flow cytometry through the signalling of CFSE. To test the regulatory activity of Tregs, 3.5 × 10^4^ CFSE‐labelled (2 μmol/L, Invitrogen, Life Technologies) CD8^+^ T cells were firstly seeded in 96‐well plates in 200 μL RPMI medium containing rhIL‐2, and then, the unlabelled CD4^+^CD25^high^ regulatory T cells isolated from patients or healthy controls were added into each well at a mixing ratio of 1:1. After co‐culturing for 7 days, the proliferation rate of CD8^+^ T cells was analysed by FACS through signalling of CFSE. The results were calculated by FlowJo software version 7.6.1 (Tree Star, Ashland, OR).

### 2.5 Elisa assay for serum cytokines

The serum levels of anti‐inflammatory cytokines including TGF‐β, IL‐10 and sCTLA‐4 were measured by Elisa kits (Xi Tang Biotechnology, Shanghai, China) according to the manufacturer's instructions. The serum level of HO‐1 and its reactive products, bilirubin, CoHb and iron (Fe^2+^) were also tested by the corresponding serum detection kits (Xi Tang Biotechnology, Shanghai, China) according to the manufacturer's instructions.

### 2.6 Hemin treatment

To assess the effect of Hemin, a HO‐1 inducer, on Treg cell function, Tregs were isolated from patients or controls and cultured in RPMI 1640 in the presence of Hemin at a concentration of 10 μmol/L for 24 hours. Tregs were then washed with PBS, and the proliferation of Tregs was detected by flow cytometry as described previously. The co‐culture system of CD8^+^ T cells and Hemin‐treated Tregs was established, and the reduction in CD8^+^ T cell proliferation rate was detected to evaluate the inhibitory capacity of Hemin‐treated Tregs.

### 2.7 Statistics

Data were presented as mean ± SD throughout the manuscript and analysed for statistical significance of differences between groups using Student's *t* test accounting to unequal variance.

## 3 RESULTS

### 3.1 Reduced Treg distribution in active vitiligo patients

**Figure 1:**
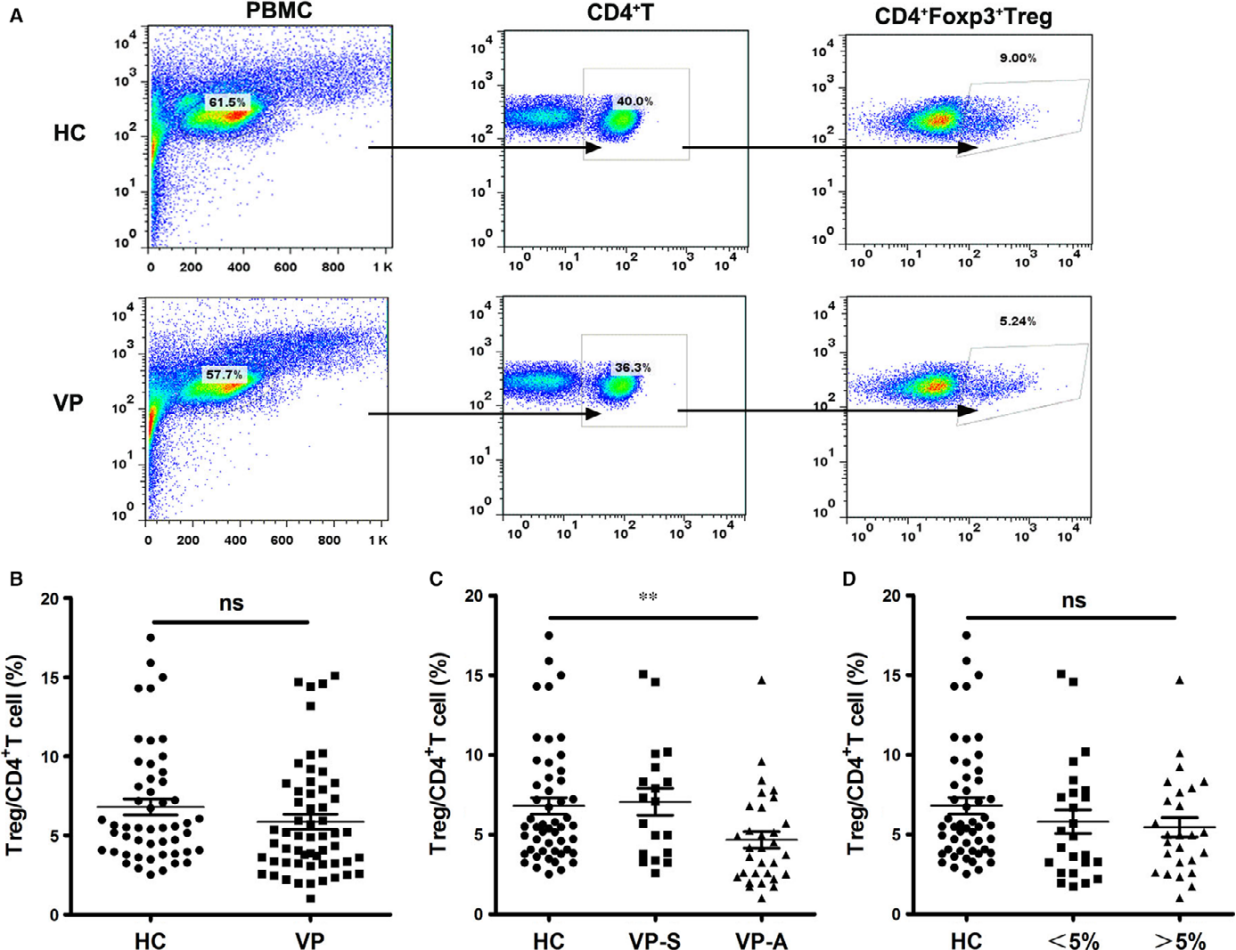
Reduced Treg distribution in active vitiligo patients. (A) Representative FACS plots for vitiligo and control blood samples, CD4^+^Foxp3^+^T cell was used to identify Tregs. (B) The average percentage of Tregs was quantified and compared among control and vitiligo samples. (C) The percentage of Tregs was analysed in healthy controls and patients in different phases. (D) The percentage of Tregs was analysed in healthy controls and patients with different lesional skin area. Values are presented as the mean ± SD, **P < .01. ns, not significant; HC, healthy controls; VP, vitiligo patients; VP‐S, stable vitiligo patients; VP‐A, active vitiligo patients

Several studies have tested the amount of Tregs in the peripheral blood of vitiligo patients, but controversial results were reported, possibly because of the limitation of sample size in each study. In consideration of this, our study collected more samples from 102 individuals consisting of 51 vitiligo patients and 51 age and sex‐matched healthy controls. Compared with healthy controls, the percentage of Tregs (CD4^+^Foxp3^+^ T cells) in the blood samples from vitiligo patients had a tendency to decrease, but with no statistical difference (Figure 1A,B). However, upon dividing the patients into two groups based on their disease activity, the percentage of Tregs showed significant decrease in active patients compared with healthy controls or stable patients, whereas there was no significant difference between healthy controls and stable patients (Figure 1C). We also compared the percentage of Tregs in patients with different lesional areas, however, with no significant difference observed (Figure 1D). Therefore, the amount of Tregs can be decreased in active vitiligo patients, rather than in stable vitiligo patients.

### 3.2 The immunoregulatory function and proliferative ability of Tregs were suppressed in vitiligo patients

**Figure 2:**
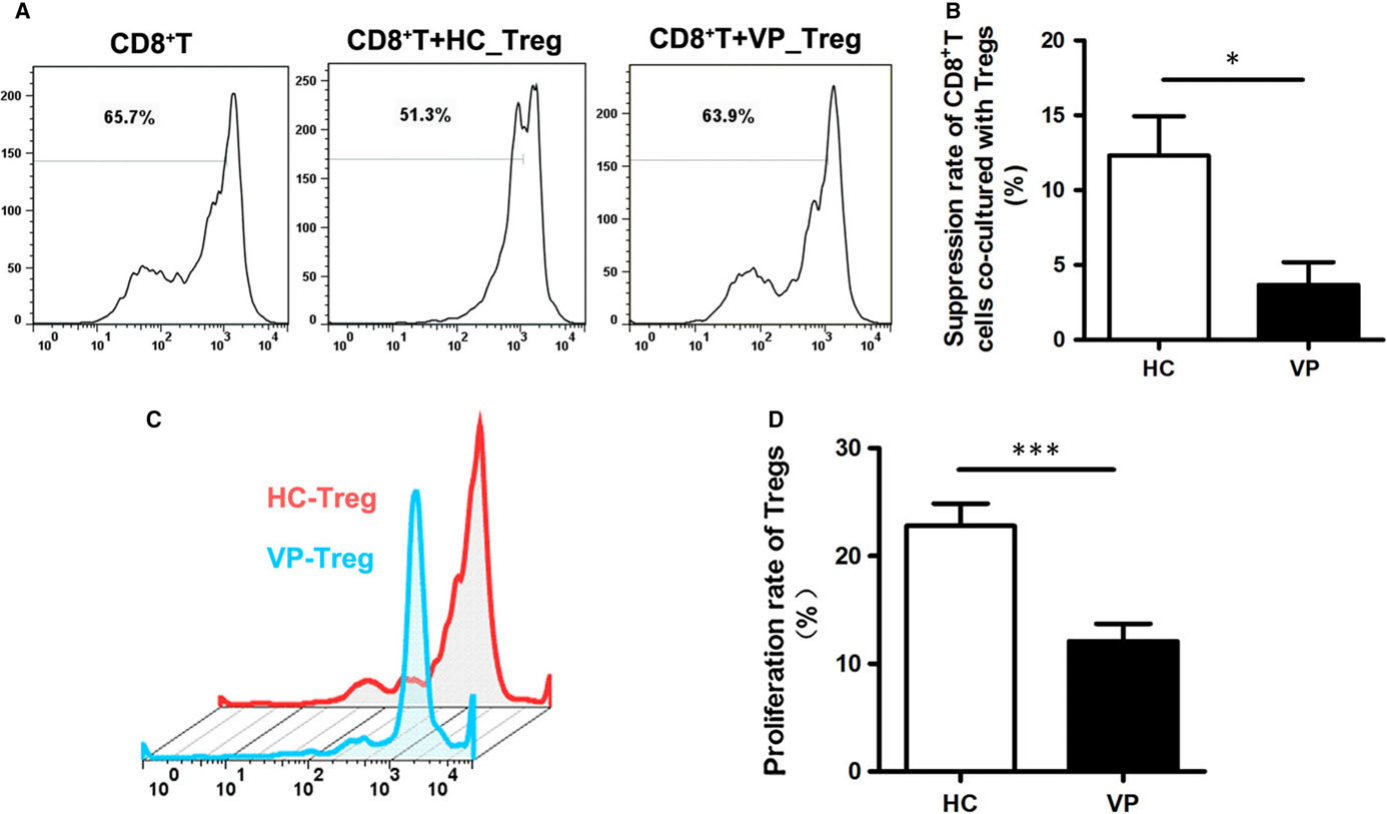
Vitiligo Tregs were impaired in the suppressive and proliferative function. (A) Representative FACS chart for Tregs suppressive ability analysis. The effector CD8^+^ T cells were stained with CFSE, the reduction in proliferation rate of effector CD8^+^ T cells was used to evaluate the suppressive function of Tregs. (B) Statistical analysis for suppressive function of Tregs in healthy controls and vitiligo patients. (C) Representative FACS chart for Tregs proliferation ability analysis. (D) Statistical analysis for proliferative function of Tregs from healthy controls and vitiligo patients. Values are presented as the mean ± SD, *P < .05, ***P < .001. HC, healthy controls; VP, vitiligo patients

The homoeostasis of the immune system depends on proper function of Treg cells. We compared the suppressive ability of Tregs towards CD8^+^ effector T cells. It turned out that the proliferation rate of effector T cells decreased more than 10% with the co‐culture of Tregs isolated from healthy controls, but only reduced less than 5% with the co‐culture of Tregs from vitiligo patients (Figure 2A,B). Therefore, the immunoregulatory effect of Tregs on CD8^+^ effector T cells was inhibited in vitiligo. Besides, the proliferation rate of Tregs from vitiligo patients was significantly lower compared with that of Tregs from healthy controls under the same culture condition (Figure 2C,D). Collectively, these data demonstrated that the immunoregulatory function and proliferative ability of Tregs were suppressed in vitiligo.

### 3.3 Immunosuppressive molecular profiles were different between vitiligo and healthy controls

**Figure 3:**
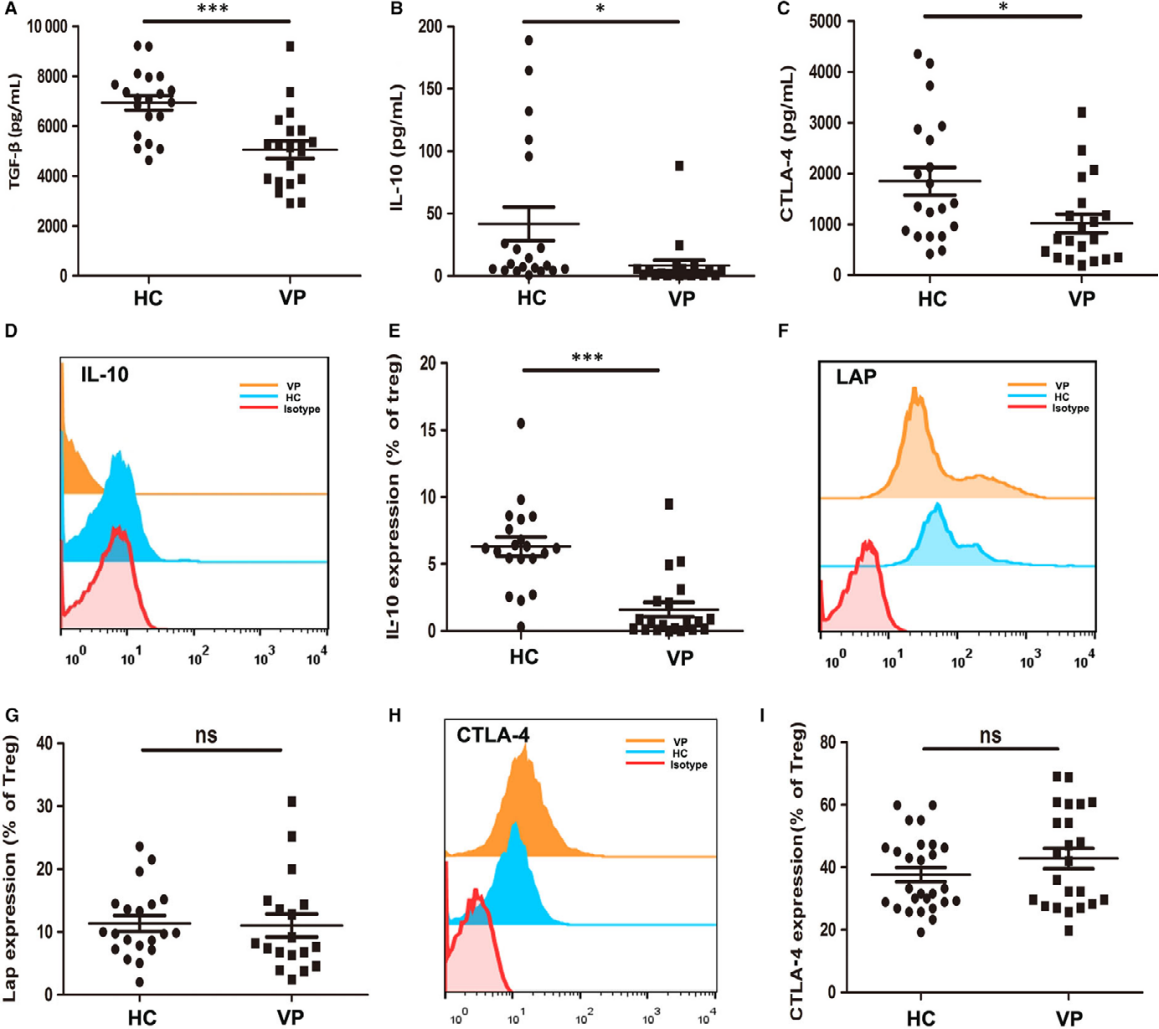
The concentrations of cytokines contributing to the plasticity of Treg cells in serum and their expression level in Treg cells. (A‐C) Elisa assay for serum levels of TGF‐β, IL‐10 and sCTLA‐4 of healthy controls and vitiligo patients (n = 20). (D and E) Flow cytometry detection of IL‐10 expression in Tregs of healthy controls and vitiligo patients (n = 20). (F and G) Flow cytometry detection of LAP expression in Tregs of healthy controls and vitiligo patients (n = 20). (H and I) Flow cytometry detection of CTLA‐4 expression in Tregs of healthy controls and vitiligo patients (n = 20). Values are presented as the mean ± SD, *P < .05, ***P < .001, ns, not significant. HC, healthy controls; VP, vitiligo patients

Producing immunosuppressive cytokines is one of the important ways for Tregs to execute their immunoregulatory function. To find out the detailed molecules responsible for the function defects of vitiligo Tregs, we tested the immunosuppressive cytokine levels in serum samples of 20 patients and 20 age and sex‐matched controls by Elisa. The results showed that serum levels of TGF‐β, IL‐10 and sCTLA‐4 significantly reduced in vitiligo patients compared with healthy controls (TGF‐β: 5054.1 ± 1558.01 pg/mL vs 6929.7 ± 1298.47 pg/mL, *P* < .001; IL‐10: 8.39 ± 19.63 pg/mL vs 41.80 ± 60.15 pg/mL, *P* < .05; sCTLA‐4: 1017.9 ± 825.87 pg/mL vs 1846.8 ± 1223.91 pg/mL, *P* < .05) (Figure 3A‐C), which indicated that vitiligo Tregs have defects in secreting immunosuppressive cytokines. We further assessed the intracellular level of these cytokines in Tregs by flow cytometry. As a result, the expression of IL‐10 significantly decreased in vitiligo Tregs than that in healthy controls (Figure 3D,E), while the expression of CTLA‐4 and latency‐associated peptide (LAP), a membrane‐bound TGF‐β complex, in Tregs was not significantly different between the two groups (Figure 3F‐I). These results suggested that decreased production of IL‐10 could be the critical factor responsible for the function defect in vitiligo Tregs.

### 3.4 The expression of HO‐1 and the levels of its reactive products were decreased in vitiligo

**Figure 4:**
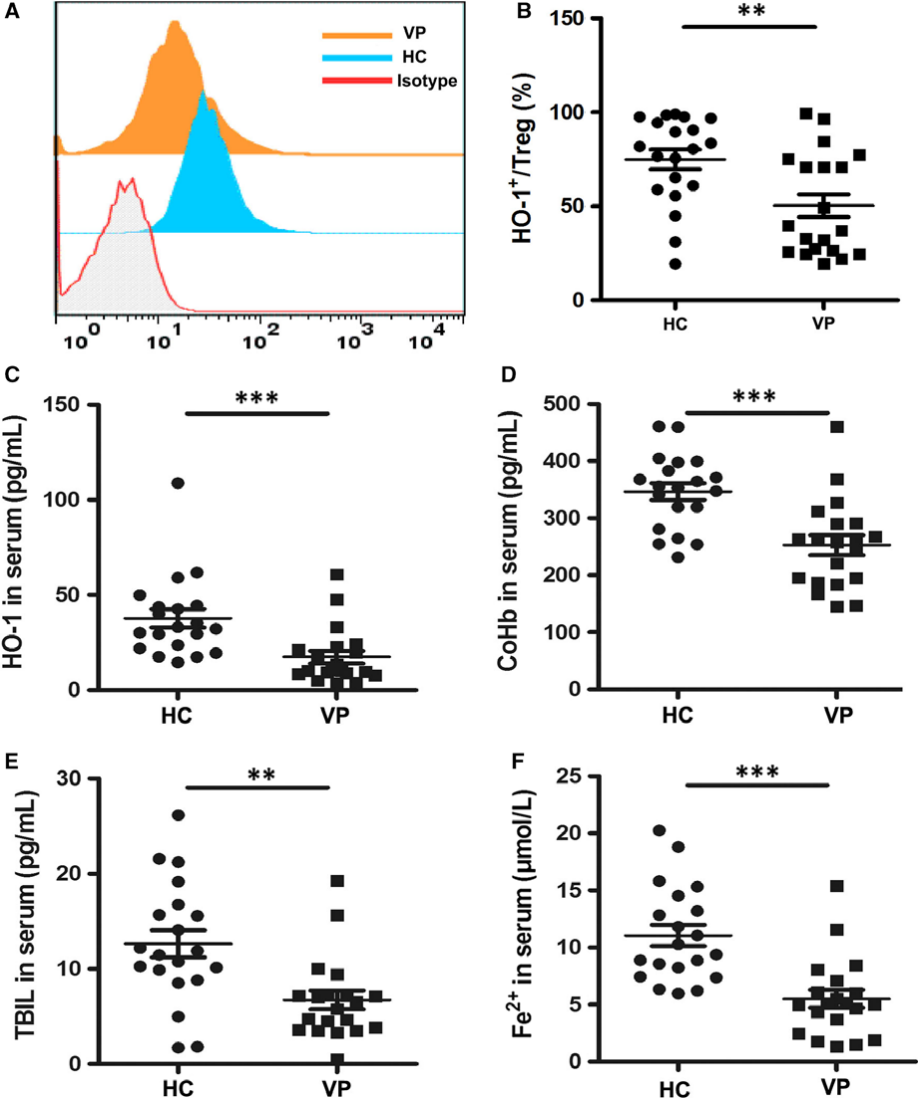
HO‐1 and its reactive products level in healthy controls and vitiligo patients. (A and B) Representative FACS plots and relevant statistical comparisons of HO‐1 expression in Tregs of vitiligo patients and healthy controls (n = 20). (C) Elisa assay for serum level of HO‐1 (n = 20). (D) Elisa assay for serum level of CoHb (n = 20). (E) Elisa assay for serum level of TBIL (n = 20). (F) Elisa assay for serum level of Fe^2+^ (n = 20). Values are presented as the mean ± SD, **P < .01, ***P < .001. ns, not significant. HC, healthy controls; VP, vitiligo patients

HO‐1 and its reactive products have been described as critical modulators for the function of Tregs. To evaluate the expression of HO‐1 in vitiligo patients, we tested the level of HO‐1 in both Tregs and serum samples from both vitiligo patients and healthy controls. Flow cytometric analysis revealed a significant decrease in the percentage of CD4^+^Foxp3^+^HO‐1^+^ T cells in vitiligo patients (Figure 4A,B). Further Elisa analysis showed that serum levels of HO‐1 and its reactive products including bilirubin, CoHb and iron (Fe^2+^) in vitiligo significantly reduced compared with healthy controls (HO‐1: 37.75 ± 21.43 pg/mL vs 17.43 ± 14.84 pg/mL, *P* = .0013; Tbil: 12.63 ± 6.36 pg/mL vs 6.74 ± 4.35 pg/mL, *P* = .0011; CoHb: 346.48 ± 64.92 pg/mL vs 252.70 ± 77.90 pg/mL, *P* = .0002; Fe^2+^: 11.06 ± 4.18 μmol/L vs 5.51 ± 3.47 μmol/L, *P* < .001) (Figure 4C‐I). Taken together, these results demonstrated that the expression and the function of HO‐1 in Tregs are suppressed in vitiligo.

### 3.5 Effects of HO‐1 modification on Tregs suppression and their proliferation capacity

**Figure 5:**
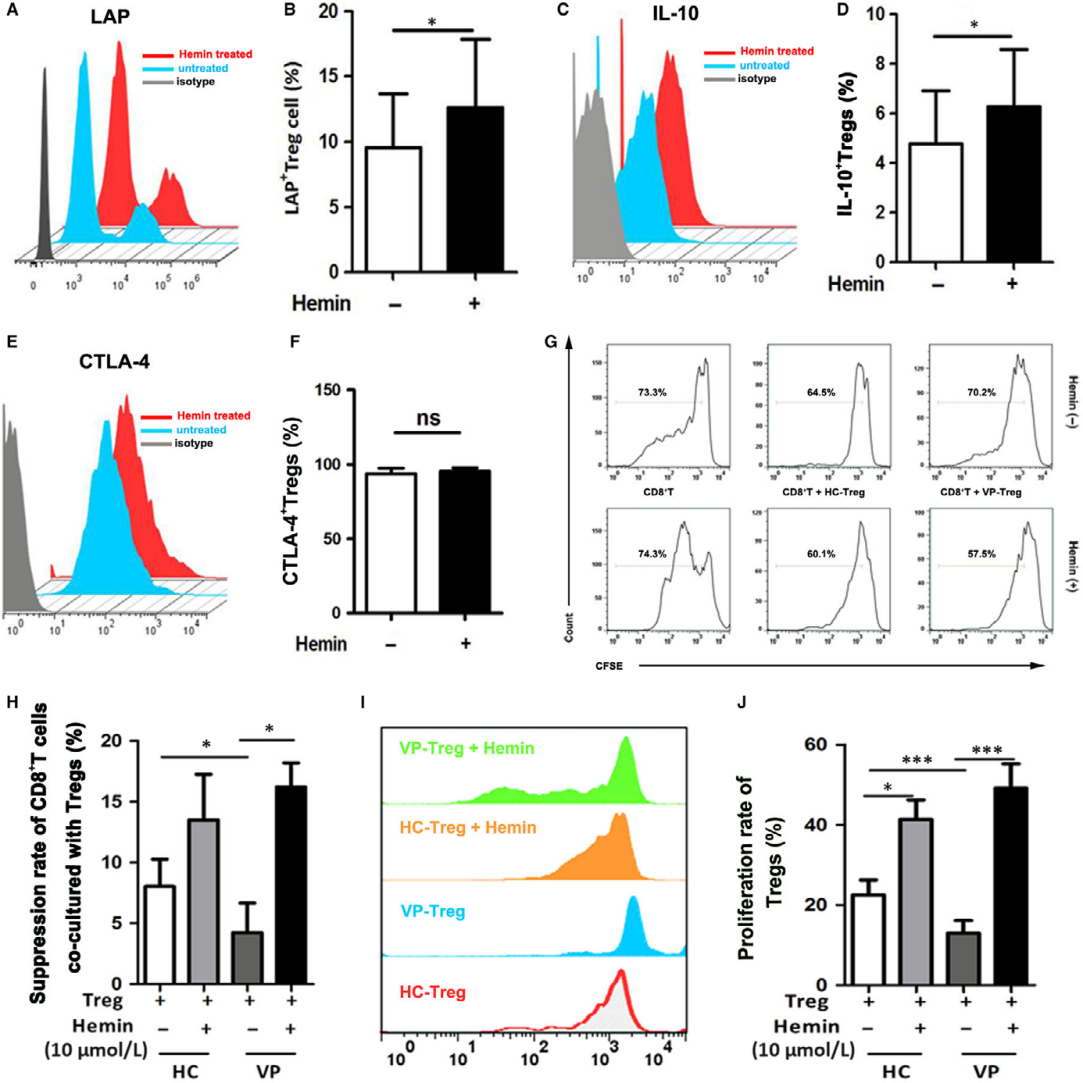
Suppressive and proliferative function of Tregs in healthy controls and vitiligo patients after HO‐1 induction by Hemin. (A and B) Representative FACS chart for LAP expression on Tregs before and after Hemin stimulation, and the relevant statistical comparisons, as indicated. (C and D) Representative FACS chart for IL‐10 expression on Tregs before and after Hemin stimulation, and the relevant statistical comparisons. (E and F) Representative FACS chart for CTLA‐4 expression on Tregs before and after Hemin stimulation and the relevant statistical comparisons. (G and H) Representative FACS chart for Treg suppressive ability analysis before and after Hemin treatment in healthy controls and vitiligo patients, and the relevant statistical comparisons. (I and J) Representative FACS chart for Treg proliferation ability analysis before and after Hemin treatment in healthy controls and vitiligo patients, and the relevant statistical comparisons. Values are presented as the mean ± SD, *P < .05, ***P < .001. HC, healthy controls; VP, vitiligo patients

Given the deficiency of HO‐1 in Tregs from vitiligo patients, we assumed that up‐regulating HO‐1 could restore the function of Tregs in vitiligo. We isolated CD4^+^CD25^high^ T cells and treated these cells with Hemin, a HO‐1 inducer and performed flow cytometry analysis. We found that the expressions of foxp3 is significantly up‐regulated(Figure S1), and correspondingly, LAP and IL‐10 were significantly up‐regulated in Hemin‐treated Tregs compared with that in untreated Tregs (Figure 5A‐D), although CTLA‐4 expression showed no alteration in both groups (Figure 5E,F), demonstrating that Hemin could promote the production of immunosuppressive cytokines including LAP and IL‐10 in vitiligo Tregs.

We further tested the suppressive effect of Tregs on effector T cells and the proliferation rate of Tregs themselves under haemin treatment. Hemin‐treated or untreated Tregs from patients or controls were co‐cultured with CD8^+^ effector T cells sorted from healthy controls, respectively. The reduced proliferation rate of effector T cells was markedly increased in Hemin‐treated group than that in untreated group. Notably, no significant difference was observed in the immunoregulatory function of Tregs between control group and vitiligo group after Hemin treatment (Figure 5G,H). These results demonstrated that Hemin is able to restore the immunoregulatory function of Tregs to normal levels in vitiligo. We went on to detect the effect of Hemin on the proliferation of Tregs. The CFSE staining showed that Hemin‐treated Tregs had a significantly higher proliferation rate in both control and patients group compared with that of untreated Tregs, with no difference between healthy Tregs and vitiligo Tregs after Hemin treatment (Figure 5I,J). Altogether, our findings suggested that enhancing HO‐1 expression could restore the immunoregulatory function of vitiligo Tregs possibly by up‐regulating IL‐10 and LAP expression.

## 4 DISCUSSION

In this study, we first found that the amount of Tregs was decreased in active vitiligo patients but not in stable vitiligo patients. Furthermore, the immunoregulatory function and proliferative ability of Tregs were suppressed in vitiligo. Moreover, the expression and the function of HO‐1 were inhibited in vitiligo Tregs. In addition, enhancing HO‐1 expression could restore the immunoregulatory function of vitiligo Tregs with up‐regulation of IL‐10 and LAP expression. Our findings indicated that HO‐1 deficiency could lead to the dysfunction of Tregs in vitiligo.

The homoeostasis of the immune system depends on the proper amount and function of Treg cells. Several studies have revealed that the number of Tregs is significantly decreased in vitiligo patients compared to controls,^6^, ^16^, ^17^ although some other studies suggest unaltered Treg amount^4^, ^18^ or even increased Treg amount in vitiligo.^5^ The controversial data may result from the limitation of sample size and individual factors like the genetic background. In this study, we found that Tregs were significantly decreased in active vitiligo patients, which, however, did not occur in stable vitiligo patients. Our results were consistent with a previous study that also reported decreased circulating Treg counts in active vitiligo patients compared with stable vitiligo patients.^6^

The function of Tregs in vitiligo has been proved impaired by multiple studies.^16^, ^17^ However, the mechanism involved in Treg dysfunction in vitiligo is still not clarified before. In our study, we found that the weakened immunoregulatory function and proliferative ability of Tregs in vitiligo was accompanied with lower expression of some anti‐inflammatory molecules including TGF‐β, IL‐10 and CTLA‐4. Meanwhile, the levels of HO‐1 and its reactive products were significantly decreased in serum samples from vitiligo patients compared with healthy controls. Our study provides insight into the nature of the immune abnormalities associated with HO‐1 and proposes a novel mechanism for the dysfunction of Tregs in vitiligo.

Restoring Tregs amount has long been regarded as a promising therapy for vitiligo. Chatterjee et al^19^ reported that a quantitative increase in Tregs by adoptive transfer controlled vitiligo progression in h3TA2 mice. Another study demonstrated that enhancing Treg recruitment by forcing overexpression of CCL22 in the skin could suppress depigmentation in vitiligo mouse model.^7^ Given that both the amount and the function of Tregs were inhibited in vitiligo, we speculated that restoring Treg function may also be effective in treating vitiligo. Previous studies have concluded that HO‐1 expression is necessary for Tregs to exert their immunoregulatory function.^20^, ^21^ Up‐regulation of HO‐1 activity in vivo can inhibit several immune response, including the proliferation of lymphocytes and cytotoxic immune cells‐mediated cytotoxicity.^22^, ^23^ In addition, induction of HO‐1 results in a significant up‐regulation of Foxp3 and TGF‐ß expression.^24^, ^25^ Our results showed that HO‐1 markedly enhanced the ability of Tregs to suppress the proliferation of CD8^+^ T cells in vitiligo. Moreover, treatment with haemin, an inducer of HO‐1, significantly restored the proliferative ability of Tregs. Taken together, our results indicate that HO‐1 could be targeted to restore the function of Tregs, which may be applied to the treatment for vitiligo.

In summary, our study demonstrated that HO‐1 is responsible for the impaired function of regulatory T cells in vitiligo patients. Although further studies are warranted to determine the involved mechanisms in more details, HO‐1 is of great potential to be used as a novel therapeutic target, offering a promising alternative to our current approaches to managing vitiligo.

## CONFLICT OF INTEREST

The authors declare no conflict of interest.
