# Supplementary figures and images for: HO‐1 regulates the function of Treg: Association with the immune intolerance in vitiligo
Source: J Cell Mol Med. 2018 Jul 5;22(9):4335–43. doi: 10.1111/jcmm.13723 (PMC6111856; doi:10.1111/jcmm.13723)

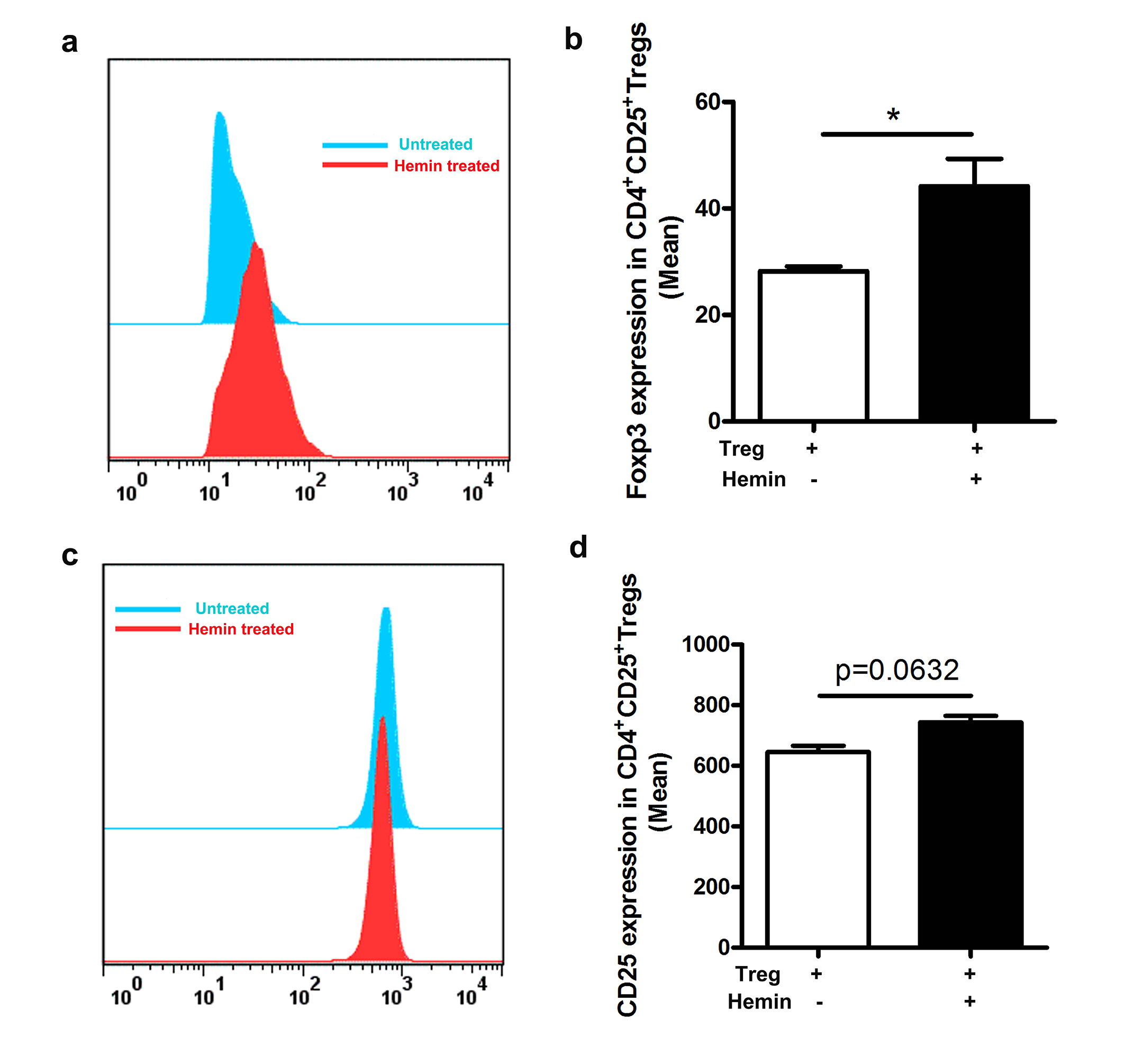

Supplement: Supplementary file 1 [file JCMM-22-4335-s001.tif]
